# Supplementary material for: Ultra-durable cell-free bioactive hydrogel with fast shape memory and on-demand drug release for cartilage regeneration
Source: Nat Commun. 2023 Nov 27;14:7771. doi: 10.1038/s41467-023-43334-8 (PMC10682016; doi:10.1038/s41467-023-43334-8)
Supplement: Supplementary file 1 — Supplementary Information [file 41467_2023_43334_MOESM1_ESM.pdf]

Supplementary Information

Ultra-durable Cell-free Bioactive Hydrogel with Fast Shape Memory and On-demand Drug Release for Cartilage Regeneration

Yuxuan Yang[1]*, Xiaodan Zhao[1], Shuang Wang[1], Yanfeng Zhang[2], Aiming Yang[3], Yilong Cheng[2,3,]* and Xuesi Chen[4]

[1] Key Laboratory of Shaanxi Province for Craniofacial Precision Medicine Research, College of Stomatology, Xi'an Jiaotong University, Xi'an 710049, China. [2] School of Chemistry, Xi’an Jiaotong University, Xi'an 710049, China. [3] Department of Nuclear Medicine, The First Affiliated Hospital of Xi'an Jiaotong University, Xi'an Jiaotong University, Xi'an, 710061, China. [4] Key Laboratory of Polymer Ecomaterials, Changchun Institute of Applied Chemistry, Chinese Academy of Sciences, Changchun 13022, China. These authors contributed equally: Yuxuan Yang, Xiaodan Zhao.

Correspondence and requests for materials should be addressed to Y.C. (E-mail: yilongcheng@mail.xjtu.edu.cn), Y. Y. (yangyuxuan@ xjtu.edu.cn)

Supplementary Table S1 Nucleotide primers used for RT-PCR

Abbreviations	Primer sequence (5'-3')
Acan	Forward: CCGCTGGTCTGATGGACACT<br>Reverse: AGGTGTTGGGGTCTGTGCAA
Sox9	Forward: GCAAACACGTTGCAAATGGC<br>Reverse: AACTCTGAAGGAGCCAAGCC
Col2	Forward: CTGGTCCTTCCGGCCCTAGA<br>Reverse: GGATCGGGGCCCTTCTCTCT

Supplementary Table S2 ICRS Macroscopic Evaluation of Cartilage Repair

Cartilage repair assessment ICRS	Points
Degree of defect repair	
In level with surrounding cartilage	4
75% repair of defect depth	3
50% repair of defect depth	2
25% repair of defect depth	1
0% repair of defect depth	0
Integration to border zone	
Complete integration with surrounding cartilage	4
Demarcating border <1mm	3
3/4 of graft integrated with surrounding	2
With a notable border >1mm width and 1/2 of graft integrated with surrounding	1
From no contact to 1/4 of graft integrated with surrounding cartilage	0
Macroscopic appearance	
Intact smooth surface	4
Fibrillated surface	3
Small, scattered fissures or cracks	2
Several, small or few but large fissures	1
Total degeneration of grafted area	0
Overall repair assessment	
Grade I: normal	12
Grade II: nearly normal	11-8
Grade III: abnormal	7-4
Grade IV: severely abnormal	3-1

Supplementary Table S3 ICRS Visual Histological Evaluation of Cartilage Repair

Feature	Points
Surface	
Smooth/continuous	3
Discontinuities/irregularities	0
Matrix	
Hyaline	3
Mixture: hyaline/fibrocartilage	2
Fibrocartilage	1
Fibrous tissue	0
Cell distribution	
Columnar	3
Mixed/columnar-clusters	2
Clusters	1
Individual cells/disorganized	0
Cell population viability	
Predominantly viable	3
Partially viable	1
<10% viable	0
Subchondral bone	
Normal	3
Increased remodeling	2
Bone necrosis/granulation tissue	1
Detached/fracture/callus at base	0
Cartilage mineralization (calcified cartilage)	
Normal	3
Abnormal/inappropriate location	0

Supplementary Table S4 OARSI scoring system

OARSI score = Grade × Stage

Tissue reaction	Grade
Surface intact	
Normal	0
Matrix: superficial zone intact, oedema and/or superficial fibrillation (abrasion), focal superficial matrix condensation	1
Cells: death, proliferation (clusters), hypertrophy, superficial zone Reaction must be more than superficial fibrillation only	
Surface discontinuity	
As above	2
+ Matrix discontinuity at superficial zone (deep fibrillation)	
± Cationic stain matrix depletion (Safranin O or Toluidine Blue) upper 1/3 of cartilage	
± Focal perichondronal increased stain (mid zone)	
± Disorientation of chondron columns	
Cells: death, proliferation (clusters), hypertrophy	
Vertical fissures (clefts)	
As above	3
Matrix vertical fissures into mid zone, branched fissures	
± Cationic stain depletion (Safranin O or Toluidine Blue) into lower 2/3 of cartilage (deep zone)	
± New collagen formation (polarized light microscopy, Picro Sirius Red stain)	
Cells: death, regeneration (clusters), hypertrophy, cartilage domains adjacent to fissures	

Erosion	
Cartilage matrix loss: delamination of superficial layer, mid layer cyst formation	4
Excavation: matrix loss superficial layer and mid zone	
Denudation	
Surface: sclerotic bone or reparative tissue including fibrocartilage within denuded surface	5
Microfracture with repair limited to bone surface	
Deformation	
Bone remodeling (more than osteophyte formation)	6

% involvement (surface, area, volume)	stage
No OA activity seen	0
< 10%	1
10-25%	2
25-50%	3
>50%	4

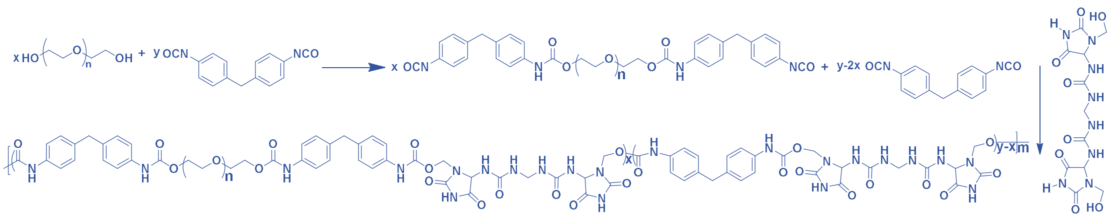

Supplementary Fig. 1 Synthetic routes of the PMI.

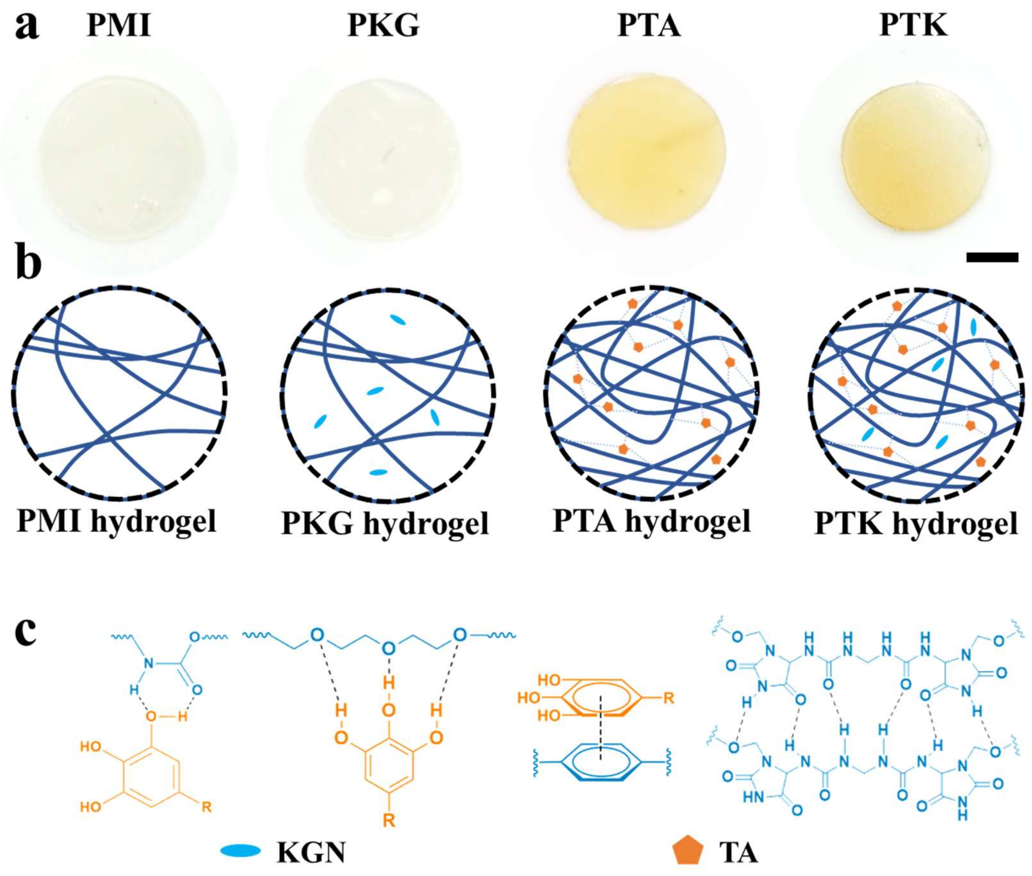

Supplementary Fig. 2 a) Photographs of PMI, PKG, PTA, and PTK hydrogels and b) illustration of the network. Scale bar: 5 mm c) The possible non-covalent interactions within PTK hydrogel.

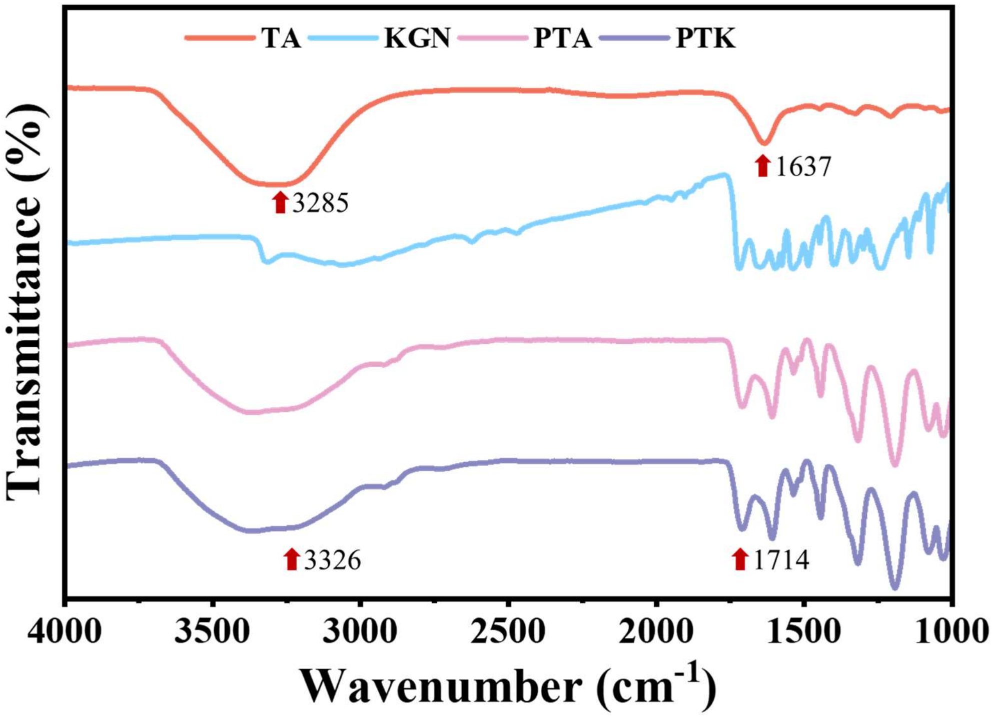

Supplementary Fig. 3 FT-IR spectra of TA, KGN, PTA, and PTK hydrogels.

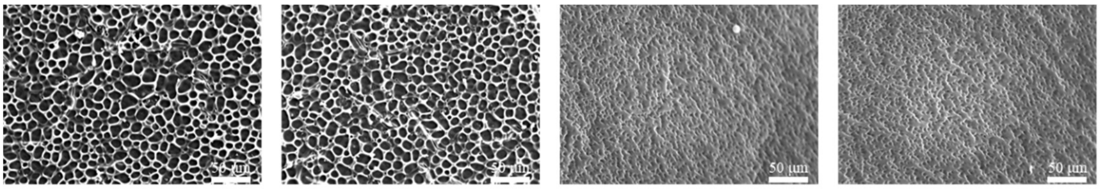

PMI PKG PTA PTK

Supplementary Fig. 4 SEM images of PMI, PKG, PTA, and PTK hydrogels.

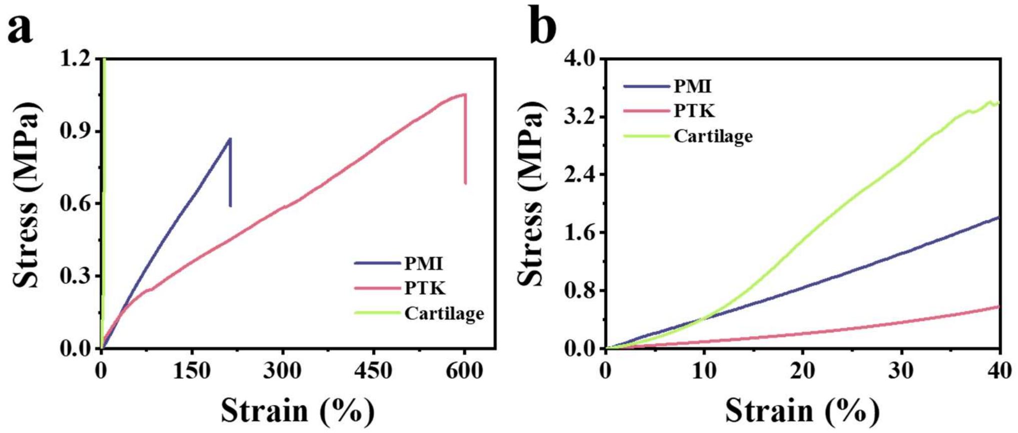

Supplementary Fig. 5 a) Tensile stress-strain curves of natural porcine cartilage, PMI and PTK hydrogels. b) Compress stress-strain curves of natural porcine cartilage, PMI and PTK hydrogels.

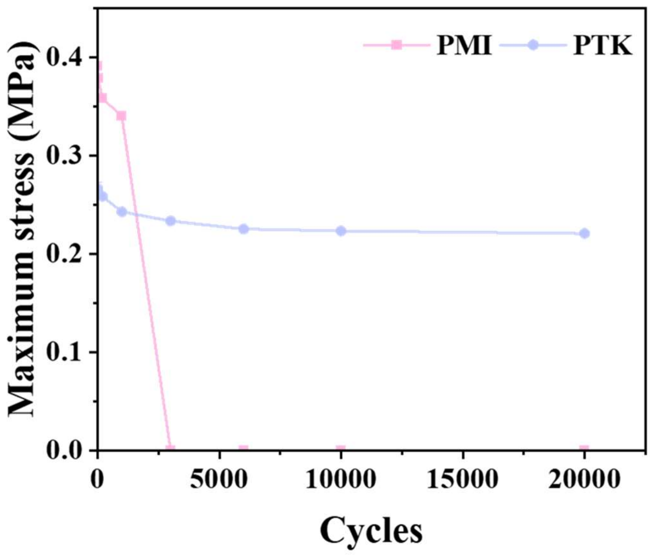

Supplementary Fig. 6 Maximum stresses of PMI and PTK hydrogels at 0, 20, 200, 1000, 3000, 6000, 10000, and 20000 cycles during the successive loading-unloading test.

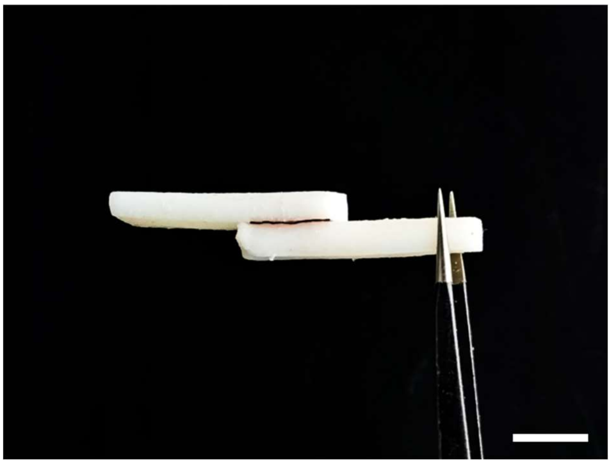

Supplementary Fig. 7 Graphic illustration of cartilage sample used in PTK hydrogel tissue adhesive test. Scale bar: 10 mm

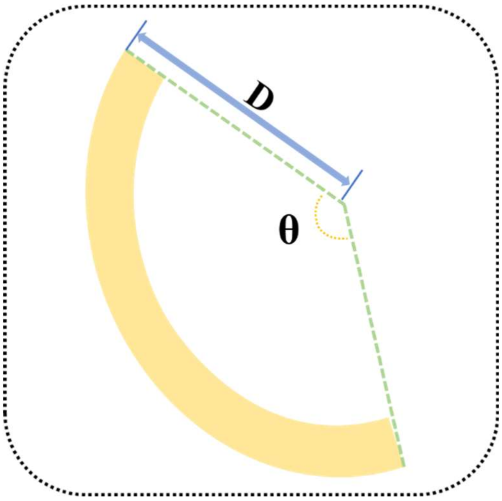

Supplementary Fig. 8 Graphic illustration of central angle (θ) and diameter (D) in shape-memory evaluation.

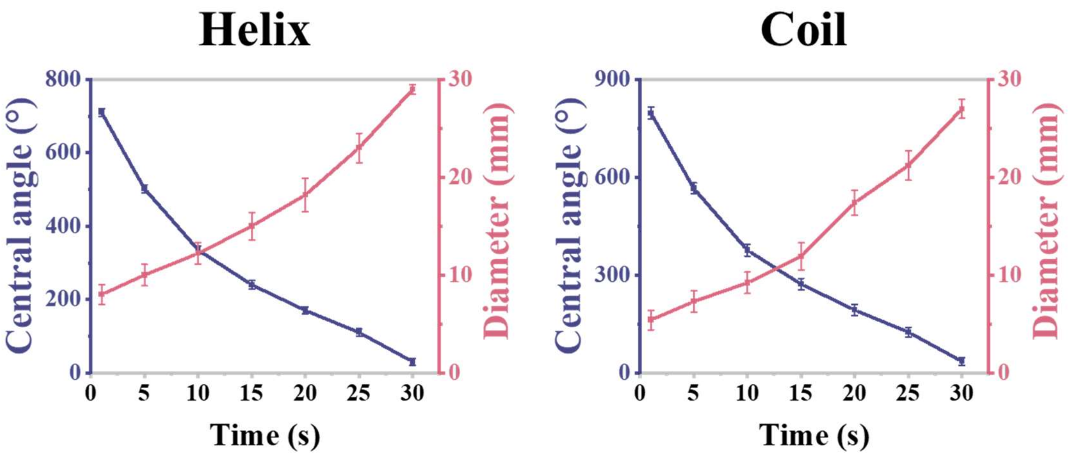

Supplementary Fig. 9 Changes of central angle and diameter during shape recovery processes. Values represent mean ± SD. Source data are provided as a Source data file. (n = 3 independent samples).

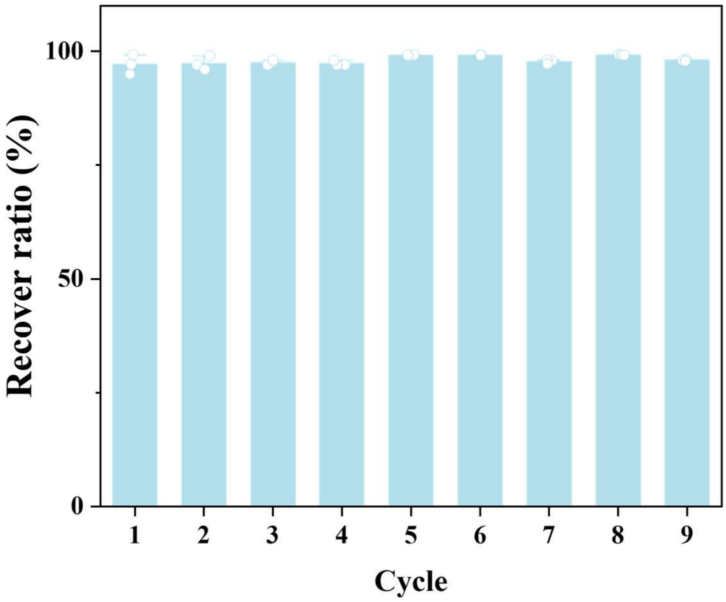

Supplementary Fig. 10 Shape recovery rate of PTK hydrogel in 9 successive shape fixation and recovery cycles. Values represent mean ± SD. Source data are provided as a Source data file. (n = 3 independent samples).

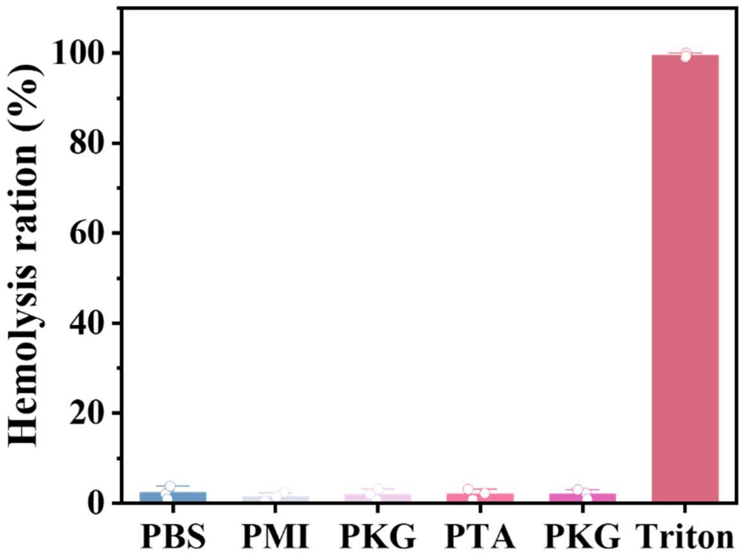

Supplementary Fig. 11 Hemolysis ratio of PBS, Triton X-100, PMI, PKG, PTA, and PTK hydrogels. Values represent mean ± SD. Source data are provided as a Source data file. (n = 3 biologically independent samples).

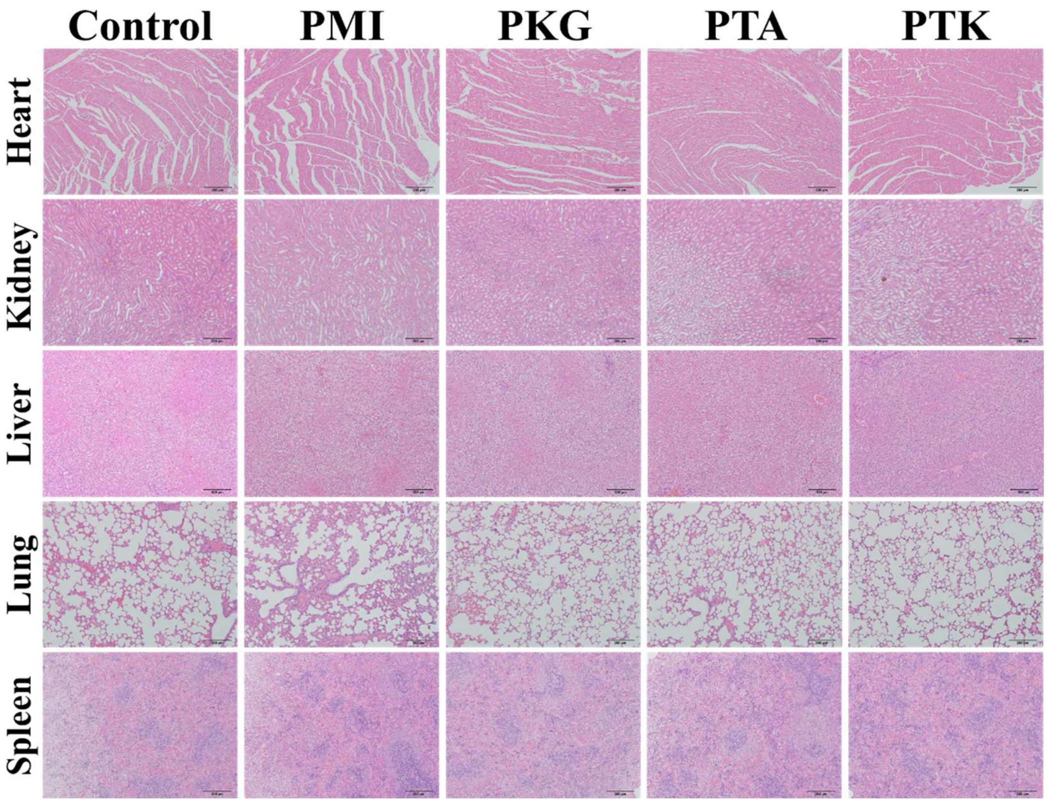

Supplementary Fig. 12 Long term in vivo biocompatibility of PMI, PKG, PTA, and PTK hydrogels. Scale bar: 200 μm.

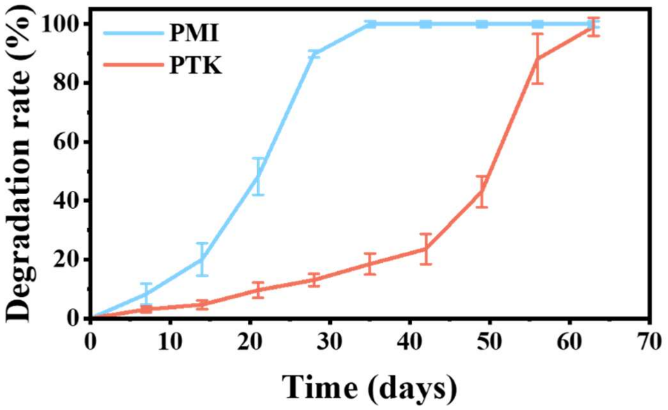

Supplementary Fig. 13 In vivo degradation behaviors of PMI and PTK hydrogels. Values represent mean ± SD. Source data are provided as a Source data file. (n = 3 independent samples).

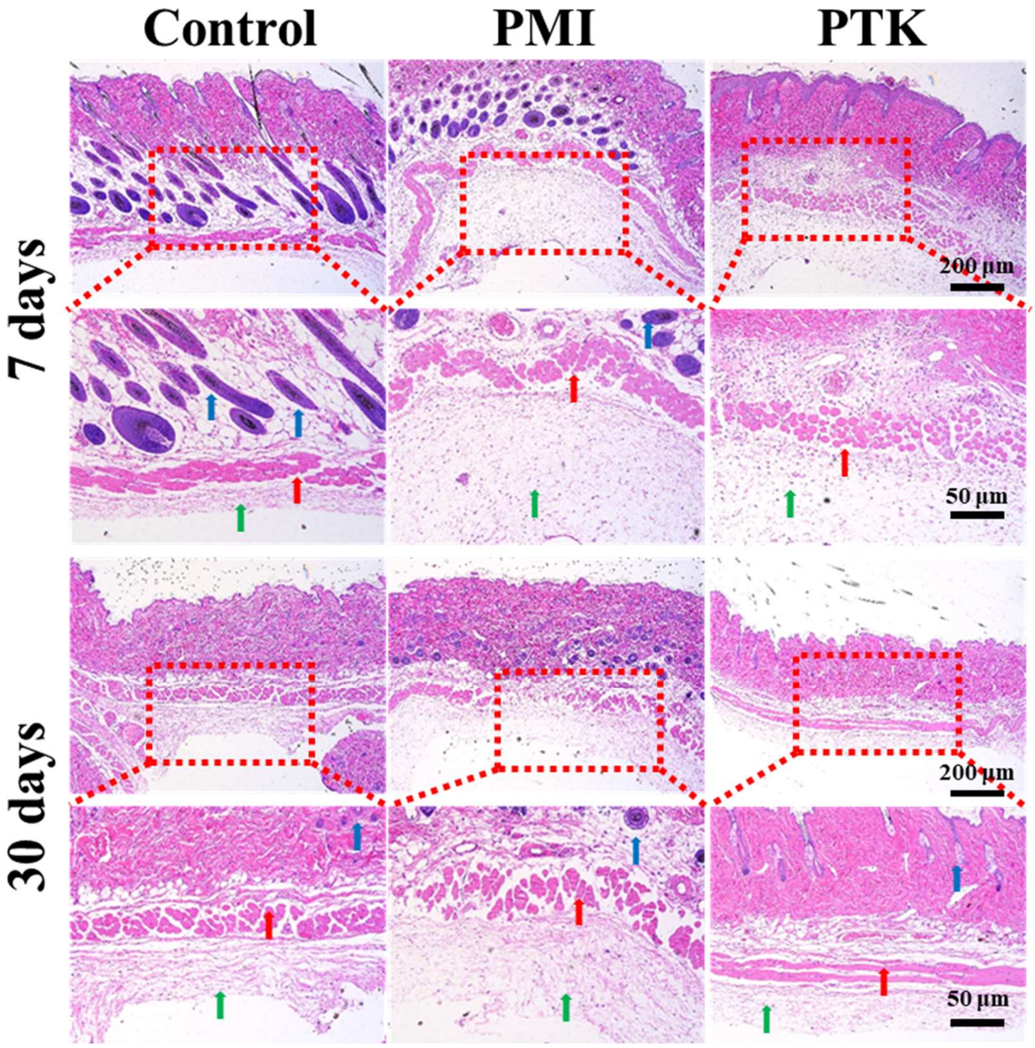

Supplementary Fig. 14 In vivo tissue biocompatibility of the hydrogels after subcutaneous implantation for 7 and 30 days. (Blue arrow: hair follicle; red arrow: muscle tissue; green arrow: subcutaneous tissue. No inflammation cell infiltration was identified.)

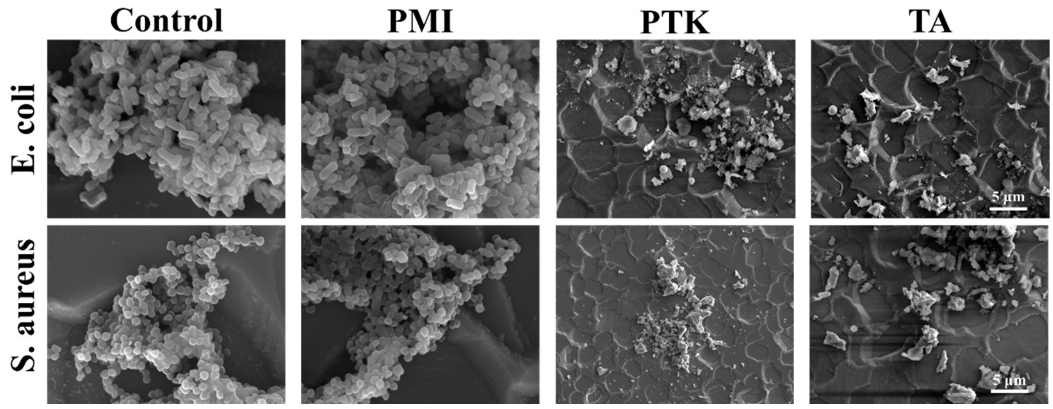

Supplementary Fig. 15 SEM images of *E. coli* and *S. aureus* with different treatments.

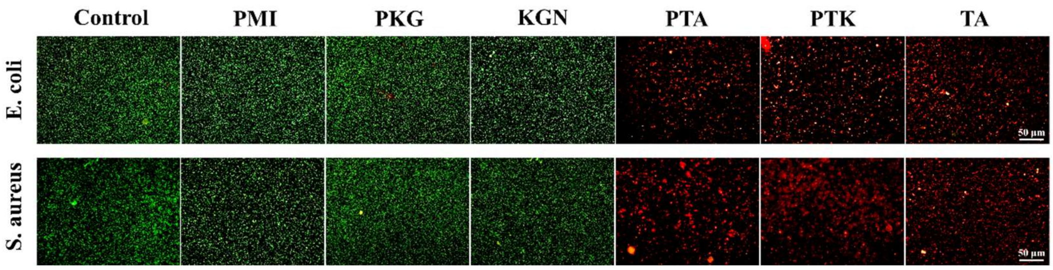

Supplementary Fig. 16 Live/Dead staining of *E. coli* and *S. aureus* with different treatments.

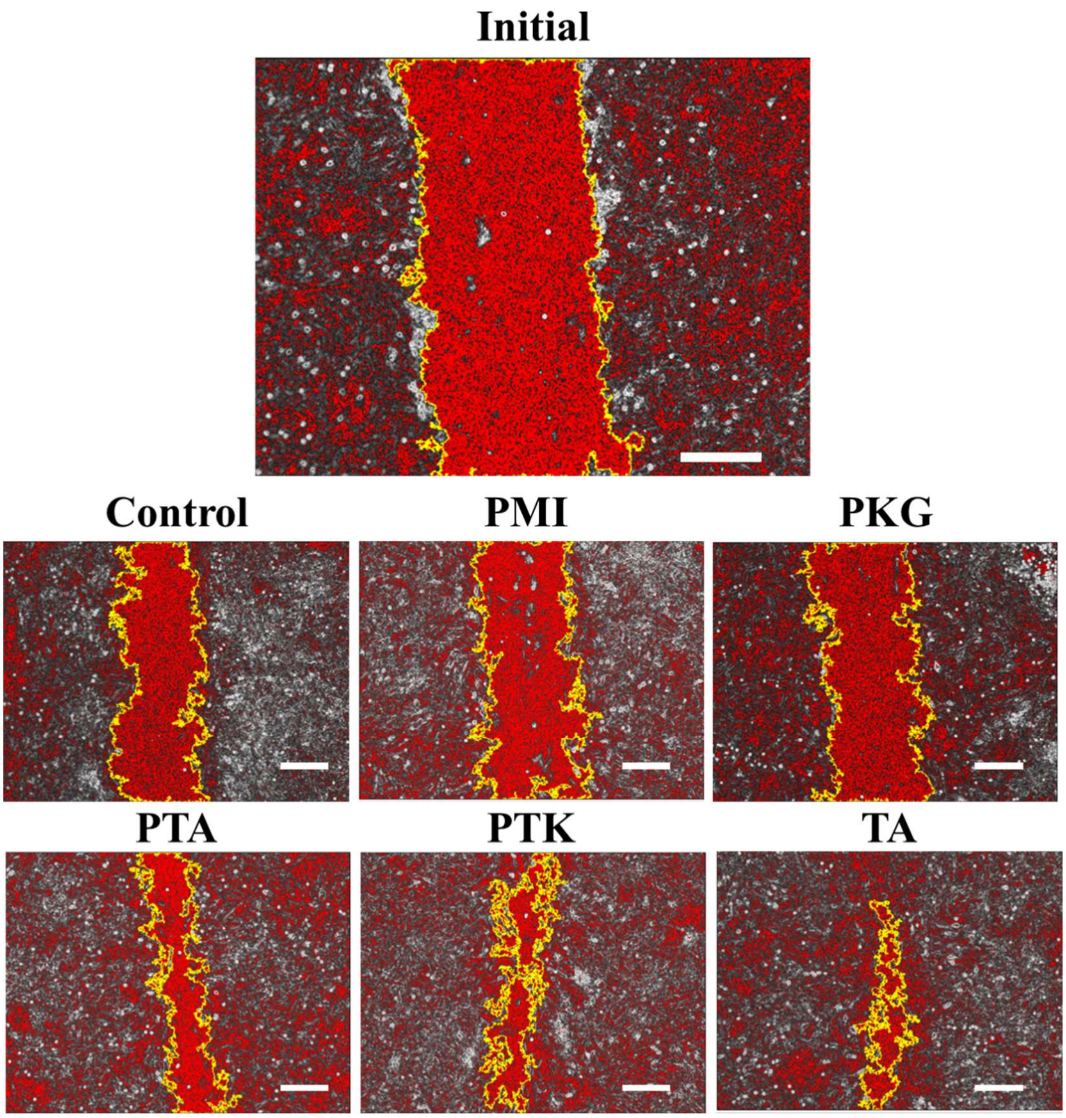

Supplementary Fig. 17 The uncovered area in cell scratch migration assay. The margin of uncovered area was identified with yellow curve by Image J software. The reduced uncovered area could reflect the migration ability of BMSCs with different treatments. Scale bar: 200 μm.

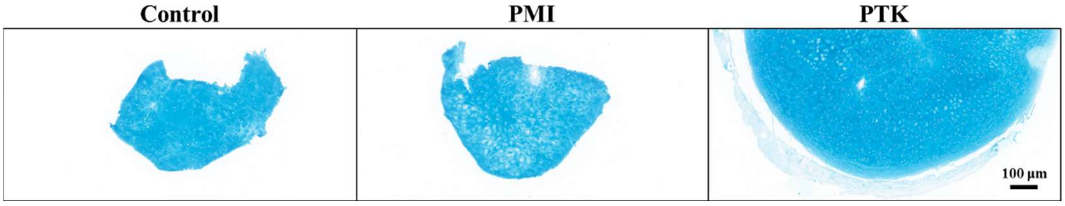

Supplementary Fig. 18 In vitro chondrogenic differentiation evaluation with different treatments.

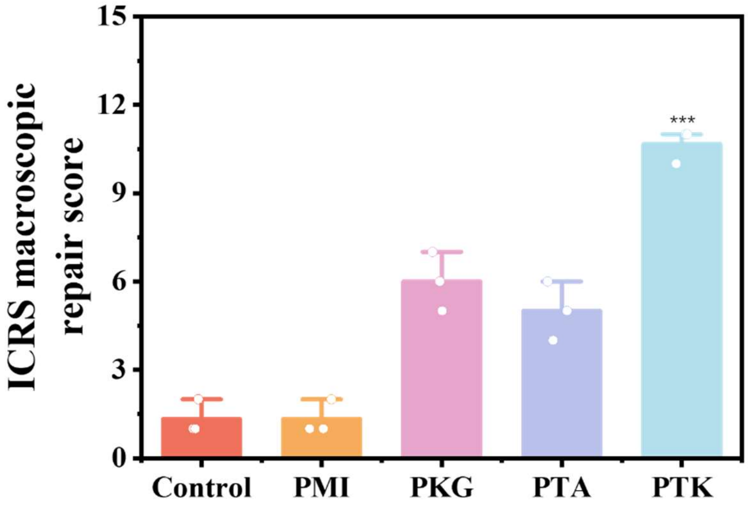

Supplementary Fig. 19 ICRS macroscopic scores of control, PMI, PKG, PTA, and PTK hydrogel-treated groups. Values represent mean ± SD. One-way ANOVA test. Source data are provided as a Source data file. (n = 3 biologically independent samples. *$P < 0.05$, **$P < 0.01$, ***$P < 0.001$).

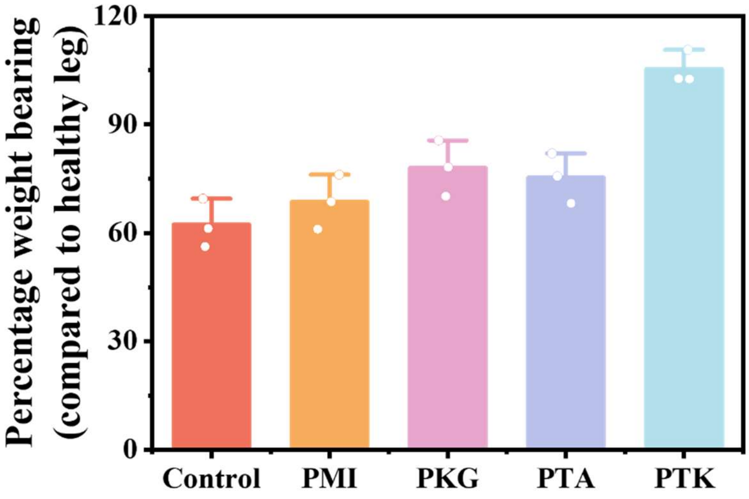

Supplementary Fig. 20 Load bearing capacities of the rats 8 weeks after surgery with different treatments. Values represent mean ± SD. Source data are provided as a Source data file. (n = 3 biologically independent samples).

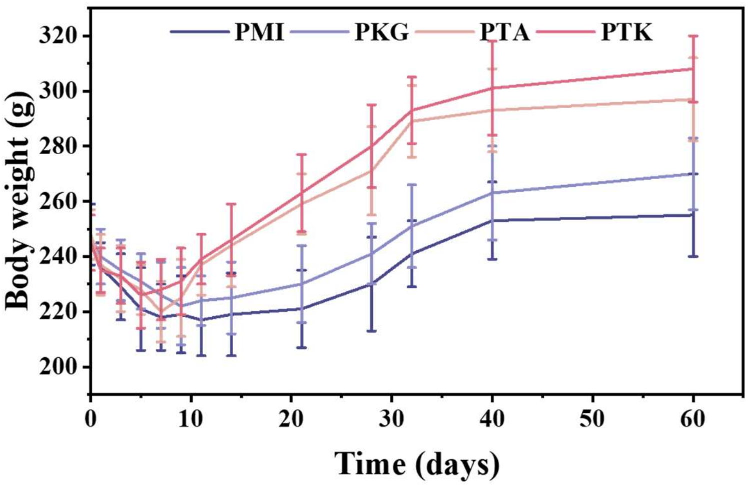

Supplementary Fig. 21 Body weight variation during experimental period with different treatments. Values represent mean ± SD. Source data are provided as a Source data file. (n = 3 biologically independent samples).

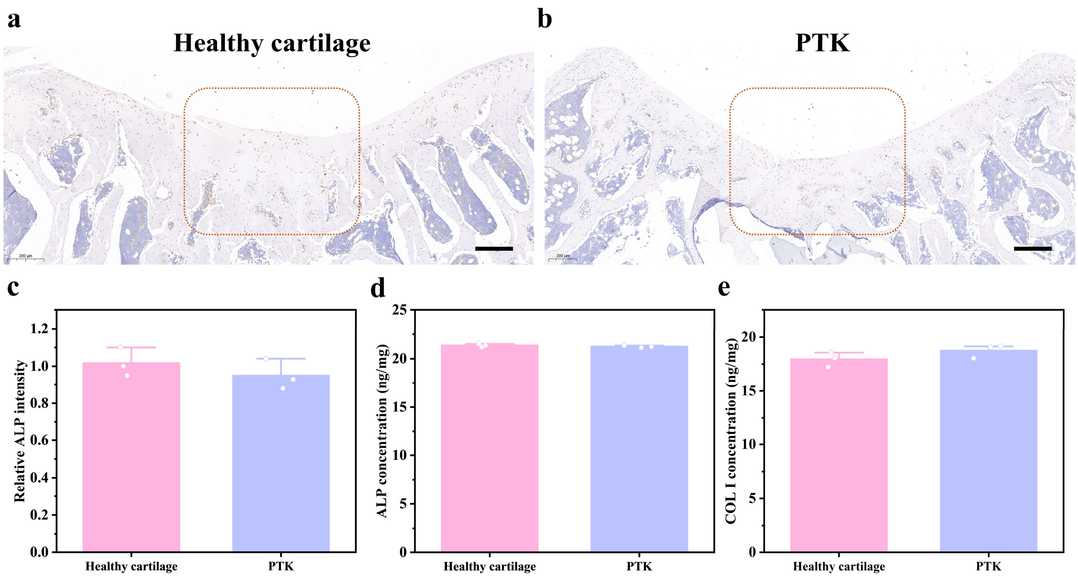

Supplementary Fig. 22 a) Immunohistochemical staining of ALP in untreated healthy cartilage. b) Immunohistochemical staining of ALP in PTK hydrogel treated group. c) Semiquantitative analysis of ALP expression in untreated healthy cartilage and PTK hydrogel treated groups based on immunohistochemical staining. Scale bar: 200 μm. d) ELISA test of ALP expression in untreated healthy cartilage and PTK hydrogel treated groups. e) ELISA test of COL I expression in untreated healthy cartilage and PTK hydrogel treated group. Values in c, d, and e represent mean ± SD. One-way ANOVA test. Source data are provided as a Source data file. (n = 3 biologically independent samples. **P* < 0.05, ***P* < 0.01, ****P* < 0.001).
