# Supplementary material for: Ultra-durable cell-free bioactive hydrogel with fast shape memory and on-demand drug release for cartilage regeneration
Source: Nat Commun. 2023 Nov 27;14:7771. doi: 10.1038/s41467-023-43334-8 (PMC10682016; doi:10.1038/s41467-023-43334-8)
Supplement: Supplementary file 3 — Description of additional supplementary files [file 41467_2023_43334_MOESM3_ESM.pdf]

Description of additional supplementary files

Supplementary Movie 1 : Demonstration of the assessment of weight bearing capacity of lower limbs in rats.
