# Supplementary material for: Ultra-durable cell-free bioactive hydrogel with fast shape memory and on-demand drug release for cartilage regeneration
Source: Nat Commun. 2023 Nov 27;14:7771. doi: 10.1038/s41467-023-43334-8 (PMC10682016; doi:10.1038/s41467-023-43334-8)
Supplement: Supplementary file 5 — Reporting Summary [file 41467_2023_43334_MOESM5_ESM.pdf]

nature portfolio

Corresponding author(s): Yilong Cheng

Last updated by author(s): Oct 5, 2023

Reporting Summary

Nature Portfolio wishes to improve the reproducibility of the work that we publish. This form provides structure for consistency and transparency in reporting. For further information on Nature Portfolio policies, see our Editorial Policies and the Editorial Policy Checklist.

Statistics

For all statistical analyses, confirm that the following items are present in the figure legend, table legend, main text, or Methods section.

n/a	Confirmed	
☐	☒	The exact sample size (*n*) for each experimental group/condition, given as a discrete number and unit of measurement
☐	☒	A statement on whether measurements were taken from distinct samples or whether the same sample was measured repeatedly
☐	☒	The statistical test(s) used AND whether they are one- or two-sided<br>*Only common tests should be described solely by name; describe more complex techniques in the Methods section.*
☒	☐	A description of all covariates tested
☒	☐	A description of any assumptions or corrections, such as tests of normality and adjustment for multiple comparisons
☐	☒	A full description of the statistical parameters including central tendency (e.g. means) or other basic estimates (e.g. regression coefficient) AND variation (e.g. standard deviation) or associated estimates of uncertainty (e.g. confidence intervals)
☐	☒	For null hypothesis testing, the test statistic (e.g. *F*, *t*, *r*) with confidence intervals, effect sizes, degrees of freedom and *P* value noted<br>*Give P values as exact values whenever suitable.*
☒	☐	For Bayesian analysis, information on the choice of priors and Markov chain Monte Carlo settings
☒	☐	For hierarchical and complex designs, identification of the appropriate level for tests and full reporting of outcomes
☒	☐	Estimates of effect sizes (e.g. Cohen's *d*, Pearson's *r*), indicating how they were calculated

Our web collection on statistics for biologists contains articles on many of the points above.

Software and code

Policy information about availability of computer code

Data collection	Thermo Fisher, Nicolet 6700, scanning electron microscopy (SU3500), CMT-1503 electromechanical tester (SUST Inc.), UV–Vis spectrophotometer (Lambda 35, PerkinElmer), HPLC system (SPD-M40, Shimadzu) , fluorescent microscope (DMi8, Leica)
Data analysis	image J 1.52v, OriginPro 2022b (64-bit) SR1 9.9.5.171 (Learning Edition), SPSS 2020.0.0

For manuscripts utilizing custom algorithms or software that are central to the research but not yet described in published literature, software must be made available to editors and reviewers. We strongly encourage code deposition in a community repository (e.g. GitHub). See the Nature Portfolio guidelines for submitting code & software for further information.

Data

Policy information about availability of data

All manuscripts must include a data availability statement. This statement should provide the following information, where applicable:

- Accession codes, unique identifiers, or web links for publicly available datasets
- A description of any restrictions on data availability
- For clinical datasets or third party data, please ensure that the statement adheres to our policy

All data available within the article or its supplementary materials. We have uploaded all Source Data files.

Research involving human participants, their data, or biological material

Policy information about studies with human participants or human data. See also policy information about sex, gender (identity/presentation), and sexual orientation and race, ethnicity and racism.

Reporting on sex and gender	This work does not involve human research
Reporting on race, ethnicity, or other socially relevant groupings	This work does not involve human research
Population characteristics	This work does not involve human research
Recruitment	This work does not involve human research
Ethics oversight	This work does not involve human research

Note that full information on the approval of the study protocol must also be provided in the manuscript.

Field-specific reporting

Please select the one below that is the best fit for your research. If you are not sure, read the appropriate sections before making your selection.

[x] Life sciences   [ ] Behavioural & social sciences   [ ] Ecological, evolutionary & environmental sciences

For a reference copy of the document with all sections, see nature.com/documents/nr-reporting-summary-flat.pdf

Life sciences study design

All studies must disclose on these points even when the disclosure is negative.

Sample size	The animal sample size was determined by previously published experiment protocols: doi: 10.1016/j.biomaterials.2021.121169. and https://doi.org/10.1016/j.actbio.2020.03.039.
Data exclusions	No data was excluded.
Replication	All experiments were performed at least in triplicate and all attempts at replication were successful.
Randomization	For the animal experiments, all rats were randomly assigned to different groups.
Blinding	All the cartilage scoring systems were scored by at least two independent, qualified researcher and by a third researcher in the event that the two scores differed by more than one point in any domain.

Reporting for specific materials, systems and methods

We require information from authors about some types of materials, experimental systems and methods used in many studies. Here, indicate whether each material, system or method listed is relevant to your study. If you are not sure if a list item applies to your research, read the appropriate section before selecting a response.

Materials & experimental systems

n/a	Involved in the study	
[ ]	[x]	Antibodies
[ ]	[x]	Eukaryotic cell lines
[x]	[ ]	Palaeontology and archaeology
[ ]	[x]	Animals and other organisms
[x]	[ ]	Clinical data
[x]	[ ]	Dual use research of concern
[x]	[ ]	Plants

Methods

n/a	Involved in the study	
[x]	[ ]	ChIP-seq
[x]	[ ]	Flow cytometry
[x]	[ ]	MRI-based neuroimaging

Antibodies

Antibodies used	Anti-SOX9 antibody EPR14335-78 abcam, Anti-Collagen II antibody EPR12268 abcam, Anti-beta Actin antibody EPR8227 abcam, Anti-Aggrecan antibody 13880-1-AP proteintech. All the antibodies listed here are also provided in the manuscript as well (with dilution).
Validation	Anti-SOX9 antibody EPR14335-78 was validated by the company using SOX9 knock out cell lines, please refer to the manufacture's description: https://www.abcam.cn/sox9-antibody-epr14335-78-ab185966.html

Anti-Collagen II antibody EPR12268 was validated by the company using Collagen II knock out cell lines, please refer to the manufacture's description: https://www.abcam.cn/collagen-ii-antibody-epr12268-ab188570.html
Anti-beta Actin ab8227 was validated by the company using knock out cell lines, please refer to the manufacture's description: https://www.abcam.cn/beta-actin-antibody-ab8227.html
Anti-Aggrecan antibody 13880-1-AP was validated by the company using knock out cell lines, please refer to the manufacture's description: https://www.ptgcn.com/products/ACAN-Antibody-13880-1-AP.html

Eukaryotic cell lines

Policy information about cell lines and Sex and Gender in Research

Cell line source(s)	The rat bone marrow mesenchymal stem cells were extracted from the 4-week-old SPF Sprague Dawley rats and cultured with DMEM medium.
Authentication	None of the cell lines used were authenticated.
Mycoplasma contamination	The cell line were not tested for mycoplasma contamination
Commonly misidentified lines (See ICLAC register)	Bone marrow mesenchymal stem cells (BMSCs)

Animals and other research organisms

Policy information about studies involving animals; ARRIVE guidelines recommended for reporting animal research, and Sex and Gender in Research

Laboratory animals	4-week-old SPF Sprague Dawley rats
Wild animals	The study did not involved wild animals.
Reporting on sex	All SD rats used in this study were male to avoid the protective effect of estrogen on articular cartilage.
Field-collected samples	The study did not involved samples collected from field.
Ethics oversight	All animal experiments in this study were performed in accordance with the ordinance of the Ethical Committee of Xi'an Jiaotong University. And the research protocols were also approved by the Animal Experimentation Ethics Committee of Xi'an Jiaotong University.

Note that full information on the approval of the study protocol must also be provided in the manuscript.
